# DNA Damage, Defective DNA Repair, and Neurodegeneration in Amyotrophic Lateral Sclerosis

**DOI:** 10.3389/fnagi.2022.786420

**Published:** 2022-04-27

**Authors:** Anna Konopka, Julie D. Atkin

**Affiliations:** ^1^Centre for Motor Neuron Disease Research, Faculty of Medicine, Macquarie Medical School, Health and Human Sciences, Macquarie University, Sydney, NSW, Australia; ^2^La Trobe Institute for Molecular Science, La Trobe University, Melbourne, VIC, Australia

**Keywords:** ALS, TDP-43, FUS, C9orf72, amyotrophic lateral sclerosis, DNA damage, DNA repair

## Abstract

DNA is under constant attack from both endogenous and exogenous sources, and when damaged, specific cellular signalling pathways respond, collectively termed the “DNA damage response.” Efficient DNA repair processes are essential for cellular viability, although they decline significantly during aging. Not surprisingly, DNA damage and defective DNA repair are now increasingly implicated in age-related neurodegenerative diseases, including amyotrophic lateral sclerosis (ALS). ALS affects both upper and lower motor neurons in the brain, brainstem and spinal cord, leading to muscle wasting due to denervation. DNA damage is increasingly implicated in the pathophysiology of ALS, and interestingly, the number of DNA damage or repair proteins linked to ALS is steadily growing. This includes TAR DNA binding protein 43 (TDP-43), a DNA/RNA binding protein that is present in a pathological form in almost all (97%) cases of ALS. Hence TDP-43 pathology is central to neurodegeneration in this condition. Fused in Sarcoma (FUS) bears structural and functional similarities to TDP-43 and it also functions in DNA repair. Chromosome 9 open reading frame 72 (C9orf72) is also fundamental to ALS because mutations in C9orf72 are the most frequent genetic cause of both ALS and related condition frontotemporal dementia, in European and North American populations. Genetic variants encoding other proteins involved in the DNA damage response (DDR) have also been described in ALS, including *FUS, SOD1, SETX, VCP, CCNF*, and *NEK1*. Here we review recent evidence highlighting DNA damage and defective DNA repair as an important mechanism linked to neurodegeneration in ALS.

## Introduction

Amyotrophic Lateral Sclerosis (ALS) is a rapidly progressing neurodegenerative disorder affecting motor neurons in the brain, brainstem, and spinal cord, which is usually fatal within 3–5 years from symptom onset. ALS overlaps significantly with frontotemporal dementia (FTD), the most common form of early-onset dementia (under 60 years of age) that primarily affects the frontal and temporal lobes of the brain. Together ALS and FTD represent a common disease spectrum with intersecting clinical, pathological, and genetic features, where ALS and FTD are at the two opposite ends (Lomen-Hoerth et al., 2002; Clark and Forman, 2006). Whilst ALS is primarily a movement disorder and FTD is a cognitive/behavioural condition, there is growing recognition that FTD signs can be present in a proportion of ALS patients and vice versa. This implies that there is clinical overlap between these two diseases. Furthermore, the same genes can be mutated and similar neuropathological features can be present in both conditions. This implies that these two disorders are part of a common disease spectrum, with pure ALS at one end of the spectrum, and pure FTD at the opposite end. Therefore, ALS with overlapping FTD symptoms (ALS-FTD) is situated in the middle of this spectrum (Lomen-Hoerth et al., 2002; Clark and Forman, 2006). Interestingly, a relationship between ALS and several forms of cancer (brain, tongue, and prostate) has been also reported (Freedman et al., 2005, 2013; Fang et al., 2013; Torgovnick and Schumacher, 2015), and aberrant DNA repair processes are strongly implicated in cancer progression. However, this relationship has not yet been explored in detail.

It is important to decipher the detailed mechanisms responsible for neurodegeneration in ALS (and FTD), so that successful therapeutics can be designed. However, there are currently few effective treatments for these conditions, offering little hope for patients and their families.

The clinical manifestations of ALS usually appear in mid-life (between 50 and 60 years of age). During aging, the cell is less able to deal with stressors such as environmental or endogenous insults, hence it is thought that impairment to normal cellular mechanisms progresses during lifespan. Ultimately in mid-life the accumulated damage therefore outweighs the ability of neurons to deal with the insults, leading to neurodegeneration and cell death.

The major pathological hallmark of ALS is the presence of misfolded protein inclusions in affected tissues. TAR DNA binding protein 43 (TDP-43), a DNA/RNA binding protein, is present in a pathological form (misfolded, aggregated, hyper-phosphorylated, and truncated) in almost all (97%) cases of ALS (Heyburn and Moussa, 2017). Fused in Sarcoma (FUS) is another DNA/RNA binding protein with striking structural and similarities to TDP-43. Recently pathological forms of FUS have been implicated as another key feature of ALS (Tyzack et al., 2019; Harley et al., 2020; Ikenaka et al., 2020). Not surprisingly, protein misfolding is strongly implicated as a disease mechanism in ALS/FTD. A long list of cellular events are also implicated in pathophysiology, involving either abnormal RNA/DNA metabolism or dysfunctional proteostasis mechanisms, including defects in protein degradation (the ubiquitin proteasome system and autophagy), trafficking [nucleocytoplasmic, endoplasmic reticulum (ER)-Golgi and axonal transport], cytoplasmic mis-localisation of normally nuclear proteins, mitochondrial dysfunction, glutamate excitotoxicity, ER stress, redox dysregulation, and apoptosis (Ravits et al., 2013; Robberecht and Philips, 2013; Gao et al., 2017; Mandrioli et al., 2020). Moreover, defects in DNA repair and induction of the DNA damage response (DDR) are increasingly implicated as disease mechanisms in ALS.

Neurons are post-mitotic so they are particularly susceptible to DNA damage, and with their high metabolic demands and oxygen consumption, they are particularly prone to oxidative DNA damage (McKinnon, 2013). In addition, given that motor neurons are large even by neuronal standards, their metabolic demands, and oxygen consumption are further increased compared to other cells and other types of neurons. It is possible that this contributes to the specific vulnerability of motor neurons to DNA damage in ALS. This implies that enhancing DNA repair or inhibiting DNA damage may offer novel therapeutic approaches for ALS in the future. However, currently no therapeutic strategies based on these mechanisms are available or have been trialled in ALS.

Whilst the number of DNA damage and repair proteins linked to ALS is steadily growing, there are still some aspects of the DDR that have not yet been associated with ALS. In this review we focus primarily on those features of the DDR that are directly or indirectly linked to ALS/FTD, both the functions of ALS-associated proteins in DNA damage and repair and the pathological characteristics related to the DDR in ALS. For a comprehensive analysis of DNA repair mechanisms, the reader is directed to several excellent detailed reviews on this topic, including those published in the recent “DNA damage repair” collection in *Nature Reviews Molecular Cell Biology* (Ray Chaudhuri and Nussenzweig, 2017; Stingele et al., 2017; Lans et al., 2019; Scully et al., 2019; Zhao et al., 2020; DNA Damage Repair, 2021; Lee and Paull, 2021)^1^. In addition, we refer readers to several other reviews about the role of reactive oxygen species (ROS) and mitochondrial-associated ER degradation in the DDR (see Nissanka and Moraes, 2018 and Kok et al., 2021).

## Amyotrophic Lateral Sclerosis

Most ALS cases arise sporadically (90–95%) whereas the remaining 10% are genetic (Mejzini et al., 2019). The greatest proportion of ALS and FTD cases are caused by hexanucleotide (GGGGCC) repeat expansions in the first intron of the *C9ORF72* gene, which are responsible for 40% of familial and 8% of sporadic ALS cases in Europe/North America (Lee et al., 2013; Ling et al., 2013; Balendra and Isaacs, 2018). In healthy individuals, up to 25 GGGGCC repeats are present, whereas ALS/FTD patients can harbour from hundreds to thousands of these repeats (DeJesus-Hernandez et al., 2011; Kim G. et al., 2020). Interestingly, the hexanucleotide expansion is bidirectionally transcribed and undergoes a non-canonical form of translation [repeat associated non-AUG (RAN) translation] which does not require a start codon (Niblock et al., 2016; Kim G. et al., 2020). This process results in the production of five different dipeptide repeat proteins (DPRs), polyGA, polyGP, polyGR, polyPA, and polyPR, that are strongly associated with toxicity (Freibaum and Taylor, 2017; Kim G. et al., 2020). In addition, toxicity can result from the repeat encoding-RNA in the form of RNA foci or other aberrant RNAs that sequester RNA-binding proteins (Freibaum and Taylor, 2017; Kim G. et al., 2020). The presence of the repeat expansion can also lead to loss of the normal cellular function of the C9orf72 protein, which is also associated with neurodegeneration in ALS/FTD *via* haploinsufficiency (Gendron and Petrucelli, 2018).

The remaining genetic causes of both ALS and FTD involve mutations in *FUS* and *TARDBP* (encoding TDP-43), which together account for ∼1% of all ALS cases, and a growing list of other genes linked to rarer familial forms (Abramzon et al., 2020; McCann et al., 2021). Mutations in superoxide dismutase 1 (*SOD1*) cause 18.9% of familial ALS cases but these are not associated with FTD (Zou et al., 2017). Several genes encoding proteins involved in the DNA damage response (DDR) have been identified in ALS or ALS/FTD patients. As well as *TDP-43* (Konopka et al., 2020) and *FUS* (Wang et al., 2013), this includes *NEK1* (Higelin et al., 2018), *CCNF* (encoding cyclin F) (Williams et al., 2016), *C21orf2* (van Rheenen et al., 2016), *SETX* (encoding senataxin) (Chen et al., 2006), *VCP* (Abramzon et al., 2020) and *SQSTM1* (Abramzon et al., 2020). *SETX* mutations are associated with a rare, autosomal dominant childhood- or adolescent-onset disease (Bennett et al., 2018) and *VCP* mutations are the cause of 1–2% of familial ALS cases (Koppers et al., 2012). Table 1 summarises the prevalence of mutations in genes linked to DNA repair that are associated with ALS.

**TABLE 1:** Prevalence of mutations present in ALS in genes encoding proteins associated with DNA repair.

Gene	Prevalence (% cases)	
	Sporadic ALS (sALS)	Familial ALS (fALS)
*TARDBP*, encoding TDP-43	<1% (Zou et al., 2017)	∼4% (Zou et al., 2017)
*FUS*	<1% (Deng et al., 2014; Zou et al., 2017)	∼4% (Deng et al., 2014; Zou et al., 2017)
*NEK1*	3–4% in European population (Cirulli et al., 2015; Brenner et al., 2016; Kenna et al., 2016; Nguyen et al., 2018)	
*C21orf72*	Rare, <1%	
*CCNF*, encoding cyclin F	1.39% (Williams et al., 2016)	0.6–3.3% (Williams et al., 2016)
*SETX*, encoding senataxin	Rare (Chen et al., 2006), <1%	
*VCP*	Rare (Koppers et al., 2012), <1%	1–2% (Johnson et al., 2010; Koppers et al., 2012)
*SQSTM1*, encoding sequestosome-1/p62	4.4% (Fecto et al., 2011)	1.8% (Fecto et al., 2011)
*HNRNPA1*	Rare (Kim et al., 2013), <1%	
*HNRNPA2/B1*	Rare (Kim et al., 2013), <1%	
*SARM1*	Rare (Gilley et al., 2021), <1%	
*PFN1, encoding profilin-1*		Rare (Brettle et al., 2019), <1%
*UBQLN2*	Rare (Huang et al., 2017), <1%	1–2% (Deng et al., 2011; Fecto and Siddique, 2011; Williams et al., 2012)
*ERBB4*	71.4% of people with respiratory onset ALS, 46.4% of people with non-respiratory onset (Al Khleifat et al., 2022)	
*SIGMAR1*	Rare (Al-Saif et al., 2011), <1%	
*GLE1*	Rare (Kaneb et al., 2015), <1%	
*SOD1*	1.2% (Zou et al., 2017)	18.9% (Zou et al., 2017)
*DAO*	Rare (Mitchell et al., 2010), <1%	
*KIAA1563/ALS2*	Rare (Hand et al., 2003)	
*C9ORF72*	7% (Chia et al., 2018)	34% (van Blitterswijk et al., 2013; Zou et al., 2017)
*ATXN2*	1.32% (Wang et al., 2014)	1.58% (Wang et al., 2014)
*MATR3*	Rare (Chia et al., 2018), <1%	Rare (Chia et al., 2018), <1%
*TBK1*	Rare (Chia et al., 2018), <1%	Rare (Chia et al., 2018), <1%
*ELP3*	Rare (Kenna et al., 2013; Couthouis et al., 2014), <1%	
*TIA1*	Rare (Mackenzie et al., 2017), <1%	∼2% (Mackenzie et al., 2017)

*List of genes based on Sun et al. (2020).*

## DNA Damage

Damage to DNA is a constant hazard to cells because nucleic acids are chemically unstable under physiological conditions. They are therefore susceptible to assault by both endogenous factors such as free radicals, and environmental sources, including ultraviolet and infrared radiation (Jackson and Bartek, 2009). As an example, DNA depurination at 37°C and pH 7.4 results in the loss of 9,000–10,000 bases per day by non-enzymatic hydrolytic cleavage of glycosyl bonds in a mammalian cell (Lindahl and Barnes, 2000). Highly specific cellular mechanisms exist to detect and repair DNA damage and thus combat these threats. Persistent DNA damage (genotoxic stress) triggers the specific signalling pathways, that comprise the DNA damage response (DDR) (Jackson and Bartek, 2009). The DDR senses and promotes specific repair of the lesion (detailed below) (Jackson and Bartek, 2009). However, if the lesion cannot be repaired, this drives cells into either apoptosis or senescence to prevent replicating the damaged genome and thus introducing mutations. Maintaining genomic integrity is therefore essential for cellular health and viability (Carusillo and Mussolino, 2020). This is true of most cells, but it is particularly important for post-mitotic neurons, that need to withstand a lifetime of insults to DNA (Iyama and Wilson, 2013). Furthermore, DNA damage accumulates during aging, which is the biggest risk factor for ALS (Agathangelou et al., 2018).

### Types of DNA Damage

DNA can be damaged in several different ways. This involves chemical addition or disruption to a base (creating an abnormal nucleotide or fragment), DNA replication errors, or a break in one or both DNA strands. Typical base alterations include alkylation, oxidation and methylation, loss of bases caused by hydrolysis, and bulky adduct formation involving covalent linkages between DNA and other reactive molecules (Chatterjee and Walker, 2017). DNA damage can also occur when crosslinking agents covalently connect two nucleotides from the same or opposite DNA strands, forming intra-strand or inter-strand crosslinks, respectively (Chatterjee and Walker, 2017).

Major forms of DNA damage are single-stranded and double-stranded breaks (SSBs and DSBs respectively), in which the phosphodiester bonds break in one or both DNA strands (Chatterjee and Walker, 2017). Single-strand breaks are more common than double-stranded breaks, but the latter are highly toxic lesions that can result in genetic instability, and thus are usually much more deleterious (Chatterjee and Walker, 2017). Furthermore, SSBs can also convert to the more harmful DSBs (Chatterjee and Walker, 2017).

Spontaneous SSBs and DSBs can result from the accumulation of R-loops, which are three-stranded nucleic acid structures consisting of a DNA:RNA hybrid with a displaced, non-template single-stranded DNA. R loops are central to several physiological cellular processes, including mitochondrial DNA replication, immunoglobulin diversification and transcription regulation (Barroso et al., 2019). However, they also constitute a major source of DNA damage and result from replication stress, the formation of SSBs and DSBs and genome instability (Garcia-Muse and Aguilera, 2019). Similarly, G-quadruplexes are another type of aberrant DNA structure that regulate basic nuclear processes but they can also trigger DNA damage, genome instability, and cell death. They are formed in nucleic acid sequences that are rich in guanine (Burge et al., 2006). These types of damage can inhibit both DNA replication and transcription and can ultimately lead to cell death. They can also result in a high incidence of mutations if not repaired effectively (Chatterjee and Walker, 2017).

### Types of DNA Repair

Each type of damage requires a specific mechanism of DNA repair and the DDR determines which repair pathway is activated (Harper and Elledge, 2007). The six major DNA repair pathways are detailed below (Chatterjee and Walker, 2017). Whilst the DDR responds to different forms of DNA damage distinctly, sometimes the pathways can overlap, such as those involved in SSB and DSB repair (Ma and Dai, 2018).

#### Base Excision Repair

Minor distortions to the DNA double helix induced by oxidation, deamination, alkylation, and loss of a nucleobase are repaired by base excision repair (BER). In this process DNA glycosylases recognise and remove the damaged base while leaving the sugar-phosphate backbone intact, creating an apurinic/apyrimidinic site (Krokan and Bjoras, 2013). DNA repair is then mediated by either (a) short-patch-repair, facilitated by Apurinic/apyrimidinic endonuclease (APE1), DNA polymerase (POL) β, DNA ligase 1 (LIG1) and a complex of DNA ligase III (LIG3) and X-ray repair cross-complementing protein 1 (XRCC1) (Chatterjee and Walker, 2017), or (b) long-patch-repair, which is mediated by APE1, POL β or POL δ/ε, flap endonuclease and LIG1 (Chatterjee and Walker, 2017).

#### Nucleotide Excision Repair

Bulky lesions generated by UV radiation, mutagens from the environment or chemotherapeutic agents, can be repaired by nucleotide excision repair (NER), of which there are two types; global genome NER (GG-NER) and transcription-coupled NER (TC-NER) (Chatterjee and Walker, 2017). In GG-NER, xeroderma pigmentosum, complementation group C (XPC), together with UV excision repair protein radiation sensitive 23 B (RAD23B), sense the presence of a single-stranded DNA (ssDNA) lesion. An endonuclease complex composed of DNA excision repair protein ERCC1, DNA repair endonuclease XPF (XPF-ER CC1) and xeroderma pigmentosum complementation group G protein (XPG), cut the damaged strand. Several replication proteins – proliferating cell nuclear antigen (PCNA), replication factor C (RFC), POL δ, POL ε or POL κ, and LIG1 or XRCC1–LIG3 – then carry out the final step of gap-filling synthesis and ligation (Chatterjee and Walker, 2017). In contrast to GG-NER, initiation of TC-NER occurs by the physical blockage of RNA polymerase II on the bulky DNA lesions. Cockayne syndrome protein A (CSA) and Cockayne syndrome protein B (CSB) are recruited to the lesion (Fousteri et al., 2006), followed by recruitment of transcription factor IIH and repair (Chatterjee and Walker, 2017).

#### Mismatch Repair

Mismatch repair facilitates the repair of base insertions, deletions, and mis-incorporation of bases that arise during DNA replication and recombination. These errors result in the formation of specific structures, insertion-deletion loops (IDLs). In humans, these lesions are first recognised by mismatch recognition proteins, heterodimers of either MutSα (MSH2/MSH6) or MutSβ (MSH2/MSH3). Then, MutL complexes are recruited to DNA to regulate excision of the mismatched bases. POL δ, RFC, high mobility group box 1 protein (HMGB1), and LIG1 coordinate the final steps involving the synthesis of new DNA (Li, 2008).

#### Single-Stranded Break Repair

Poly(ADP-ribose) polymerase 1 (PARP1) is an abundant nuclear protein with several functions in the DDR, including repair of both SSBs and DSBs (Ray Chaudhuri and Nussenzweig, 2017). One of the earliest events in DNA repair is the recruitment of PARP1 to various types of DNA lesions (Ray Chaudhuri and Nussenzweig, 2017). Oxidative damage to DNA, erroneous activity of DNA topoisomerase 1 (TOP1), collapse of DNA replication processes and stalled transcription events generate SSBs, which are detected by PARP1 (Davidovic et al., 2001). APE1, polynucleotide kinase 3′-phosphate (PNKP), aprataxin (APTX) and Flap endonuclease (FEN1) then prepare the ssDNA gap for filling and ligation (McKinnon and Caldecott, 2007). POL β and POL δ/ε mediate filling, while LIG1 is responsible for DNA ligation (McKinnon and Caldecott, 2007; Chatterjee and Walker, 2017).

#### DSB Repair: HR and NHEJ

DSBs are repaired by two major distinct pathways, homologous recombination (HR) and non-homologous end joining (NHEJ). However, in neurons, NHEJ is the major pathway, although it is more error prone than HR (Thompson and Schild, 2001; Lieber, 2008). In NHEJ, a specific kinase, DNA protein kinase (DNA-PK), is recruited to DSBs where it ligates the ends of DNA together with no regard for sequence homology. This can therefore generate deletion or insertion mutations (Lieber, 2008). In contrast, HR uses a specific DNA template to repair the DSB, leading to reconstitution of the original sequence. Hence, HR has much less chance of introducing mutations than NHEJ (Thompson and Schild, 2001).

In both pathways, DSBs are recognised by ataxia telangiectasia mutated kinase (ATM), which phosphorylates histone H2AX over a mega-base region of DNA surrounding a DSB. Phosphorylated H2AX (γH2AX) forms discrete foci in the nucleus that are clearly visible by microscopy, and thus are a widely used marker of the presence of DSBs (and hence DNA damage) in cells (Larry and Charles, 2000–2013). In NHEJ, the broken DNA ends are bound by parts of the DNA-PK complex, first a Ku70/Ku80 heterodimer, followed by recruitment of the catalytic subunit of DNA-PK (DNA-PKcs) (Ciccia and Elledge, 2010). These ends are then processed by the nuclease Artemis and DNA polymerases Polμ or Polλ, to create compatible ends (Lieber, 2010) that are ligated by a large DNA ligase complex involved in NHEJ. This complex contains DNA ligase 4, X-ray cross-complementation group 4 (XRCC4) and the XRCC4 like factor (XLF)/Cernunnos (Ahnesorg et al., 2006; Buck et al., 2006). Histone deacetylase 1 (HDAC1) is also implicated in the binding of PARP1 at DSBs during NHEJ (Robert et al., 2016). HDACs catalyse the removal of acetyl groups from the amino-terminal lysine residues of histones, and acetylation/deacetylation is a dynamic process that modulates several aspects of DSB repair (Li et al., 2020).

In contrast, in HR the broken ends undergo resection by the MRN-complex, CtBP-interacting protein (CtIP), and other endonucleases, which generate 3′-ss DNA (Sartori et al., 2007; Brandsma and Gent, 2012). The ssDNA tail then becomes coated with replication protein A (RPA), which subsequently becomes displaced by RAD51 to form a nucleoprotein filament. RAD51, whose function is controlled by BRCA2, searches for a homologous sequence on the sister chromatid and catalyses invasion of the strand. The recombination intermediates are then resolved (Pardo et al., 2009; Brandsma and Gent, 2012).

## The Role of Proteins Associated With ALS/FTD in the DDR

Recently, the functions of TDP-43 and FUS have been expanded to include their roles in DNA repair and the DDR. Given that the role of DNA damage in ALS is only just emerging, it is likely that future studies will reveal an even stronger relationship between these proteins, ALS, and DNA damage/repair. It is also possible that the list of proteins associated with ALS involved in the DDR will be extended in the future. Both TDP-43 and FUS are thought to function in both the detection of DSBs via sensing of γH2AX, and in their repair by NHEJ (Wang et al., 2013; Hill et al., 2016; Konopka et al., 2020). Similarly, both proteins are involved in the assembly of DNA repair complexes by creating a scaffold for the recruitment of other DNA repair proteins, such as XRCC1 or XRCC4 (Wang et al., 2018; Guerrero et al., 2019). Similarly, there is also evidence that senataxin and p62 are involved in the repair of DSBs (Hewitt et al., 2016; Rawal et al., 2020). FUS is also implicated in the repair of oxidatively damaged bases in BER (Wang et al., 2018). Senataxin, FUS and TDP-43 are implicated in the repair of R-loop associated DNA damage (Hill et al., 2016; Giannini et al., 2020). In addition, both C21orf21 and NEK1 interact with each other during DNA repair (Lai et al., 2011). We discuss these mechanisms in detail below.

### TDP-43 Participates in Transcription-Induced DNA Damage and NHEJ

TDP-43 was originally identified as a transcriptional repressor binding to the *trans*-activation response (TAR) element of HIV human immunodeficiency virus (Lee et al., 2011). The RNA binding functions of TDP-43 are well characterised, and involve many RNA metabolic processes, including transcription, splicing, and miRNA biogenesis (Lee et al., 2011). However, the DNA-binding functions of TDP-43 have been described more recently. A global proteomic study first provided evidence that TDP-43 interacts with Ku70 in HEK-293 cells, implying it may have a role in DNA repair (Freibaum et al., 2010). A subsequent report revealed that TDP-43 functions in the repair of transcription-associated DNA damage. Induction of DNA damage enhanced the co-localisation of TDP-43 with damaged DNA, as detected by the formation of γH2AX foci, and increased the co-localisation of TDP-43 with RNA polymerase II, which transcribes DNA into mRNA (Hill et al., 2016). Furthermore, depletion of TDP-43 resulted in increased sensitivity to the RNA polymerase II inhibitor α-amanitin, a transcription stalling agent, leading to the formation of more DNA breaks in U2OS cells (Hill et al., 2016). As DNA is damaged when transcription is obstructed, this implies that TDP-43 is required for DNA repair. Similarly, the accumulation of DNA damage during transcription is linked to the formation of potentially harmful R-loops (Sebastian and Oberdoerffer, 2017). Interestingly, depletion of TDP-43 enhances the formation of R loops and production of DSBs in non-neuronal HeLa cells (Giannini et al., 2020). In contrast, resolving the formation of R-loops by digestion of DNA-RNA hybrids with RNASEH1 prevented the accumulation of DNA damage induced by TDP-43 depletion (Hill et al., 2016). This implies that TDP-43 is specifically associated with the repair of DNA damage arising from the formation of R-loops. However, it does not exclude the possibility that TDP-43 also prevents the formation of R-loops itself. The precise mechanisms involved in this process therefore require further investigation. Nevertheless, one possibility is that TDP-43 resolves transcription-associated DNA damage by NHEJ DSB repair, given that recent studies have highlighted its role in this mechanism (Mitra et al., 2019; Konopka et al., 2020).

TDP-43 is recruited specifically to sites of DNA damage (Konopka et al., 2020) during NHEJ, where it acts as a scaffold for recruitment of the XRCC4-DNA ligase IV complex (Mitra et al., 2019). Knock down or knock out of TDP-43 by either shRNA or CRISPR/Cas9 techniques impairs NHEJ, resulting in the accumulation of genomic DSBs (Mitra et al., 2019). Interestingly, knockdown of TDP-43 also inhibits the formation of γH2AX foci in NSC-34 cells, suggesting that TDP-43 may be involved in the recognition of DSBs (Konopka et al., 2020).

**FIGURE 1:**
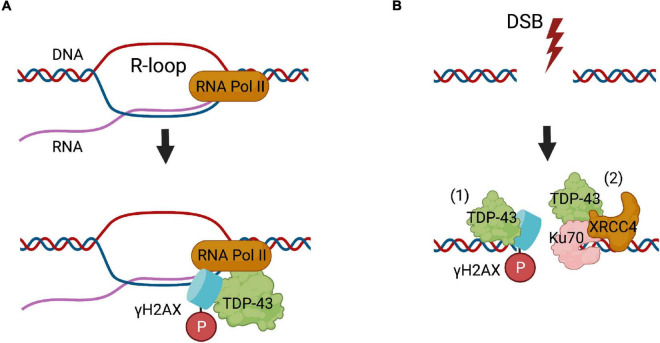
The roles of TDP-43 in DNA repair. **(A)** TDP-43 functions in the repair of R-loops formed during transcription, where it binds to γH2AX foci and RNA polymerase II (RNA Pol II). **(B)** In NHEJ DSB repair, (1) TDP-43 binds to damaged DNA, where it possibly facilitates the phosphorylation of histone H2AX. (2) TDP-43 binds to Ku70 and creates a scaffold for recruitment of the main DNA ligase involved in NHEJ, the XRCC4-DNA ligase IV complex (XRCC4).

Knockdown of TDP-43 also leads to chromatin instability and impaired DNA repair in HEK 293T cells (Kawaguchi et al., 2020). Furthermore, the interaction between TDP-43 and multiple binding partners are altered following DNA damage in HEK293T cells. Differential interactions, both increased and decreased, with proteins of the nuclear RNA exosome and ribosome, chromatin-associated proteins, transcription-coupled DNA repair proteins (Kawaguchi et al., 2020), and several important DNA repair proteins, including TOP1, Ku80, replication factor C subunit (RFC3) and NPM1 (a multi-functional protein in the DDR) have been described (Kawaguchi et al., 2020). Together, these findings provide compelling evidence that TDP-43 functions in the DDR (Figure 1).

### FUS Participates in Transcription Induced DNA Damage and NHEJ

Given the striking structural and functional similarities with TDP-43, it is not surprising that FUS is also implicated in comparable DNA repair processes, which were described prior to those involving TDP-43 (Hill et al., 2016). Similar to TDP-43, it was proposed that FUS functions in the repair of transcription associated DNA damage. Furthermore, FUS localises at sites of transcription-associated DNA damage during R loop formation (Hill et al., 2016). Depletion of FUS results in increased sensitivity to α-amanitin, leading to DNA damage due to impaired transcription, implying that FUS is required for the repair of DNA damage during transcription (Hill et al., 2016). Also similar to TDP-43, FUS either prevents the formation of R-loops or repairs the damage associated with R-loop formation. This was demonstrated by the resolution of R-loops by digestion of DNA:RNA hybrids with RNASEH1, which prevented the accumulation of DNA damage (Hill et al., 2016). Given the similarities between FUS and TDP-43, as well as the known role of FUS in NHEJ repair, it is therefore likely that FUS cooperates with TDP-43 during NHEJ to repair DNA lesions formed during transcription. However, this has not yet been confirmed experimentally.

A role for FUS in NHEJ has been also reported from its direct interaction with HDAC1 (Wang et al., 2013). Interestingly, induction of DNA damage by etoposide enhances this interaction, and both proteins are necessary for successful DNA repair (Wang et al., 2013). Furthermore, knockdown of FUS diminishes γH2AX expression and resulted in less accumulation of DDR factors, nibrin (NBS1), pATM, and Ku70 at DSB sites, suggesting that FUS functions in one of the earliest stages of the DDR (Wang et al., 2013). FUS is also recruited to UVA irradiation-induced DNA damage in a PARP-dependent manner (Mastrocola et al., 2013; Rulten et al., 2014; Altmeyer et al., 2015). In addition, the interactions of FUS with Ku80 and NPM1 are also altered following DNA damage, highlighting the role of FUS in the repair of DNA breaks (Kawaguchi et al., 2020).

**FIGURE 2:**
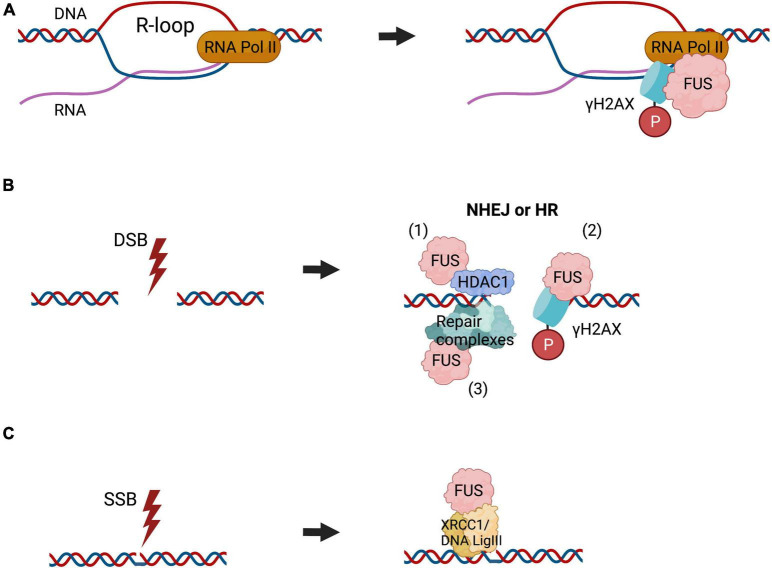
The roles of FUS in DNA repair. FUS is implicated in diverse DNA repair pathways. **(A)** FUS functions in the repair of R-loop associated DNA damage, formed during transcription, where it binds to γH2AX foci and RNA polymerase II (RNA Pol II), similar to TDP-43. **(B)** FUS is recruited to the sites of DSBs, which can be repaired by NHEJ or HR, although in neurons NHEJ is the most important mechanism. FUS interacts directly with HDAC1 to regulate DSB repair (1). It also participates in the phosphorylation of histone H2AX (2) and likely facilities the assembly or stabilisation of DNA repair complexes (3). **(C)** FUS is also implicated in BER DNA repair, by facilitating the assembly or stabilisation of DNA repair complexes. This also includes recruitment of the XRCC1/DNA ligase III α to repair oxidatively damaged bases.

Other studies have suggested a role for FUS in BER, which has not yet been described for TDP-43. In healthy neurons, the glycine-rich region of FUS (residues 268–355) facilitates PARP-1-dependent recruitment of XRCC1/DNA Ligase IIIα (LIG3) to sites of oxidatively modified bases during BER, resulting in its activation (Wang et al., 2018). Together these findings indicate that FUS has broad functions in several DNA repair pathways (Figure 2).

### Other Proteins Associated With ALS With Functions in the DNA Damage Response: NEK 1, C21orf2, Cyclin F, Senataxin, VCP, and p62

NEK1 represents one of 11 members of the highly conserved NIMA kinase family, which has functions in cell-cycle progression and mitosis (Shalom et al., 2008). Knockdown of NEK1 using stable RNA interference results in delayed DNA repair in response to diverse DNA damage-inducing agents. These include methyl-methanesulfonate, which stalls replication forks, hydrogen peroxide, which generates apurinic or apyrimidinic sites, and SSBs and DSBs, or cisplatin, which results in the formation of DNA intra- and inter-strand cross links (Pelegrini et al., 2010). This implies that NEK1 has a role in the cellular response to genotoxic stress (Pelegrini et al., 2010).

C21orf21 interacts with NEK1 and is also thought to function in DNA repair (Lai et al., 2011). C21orf21 inhibits cellular proliferation following DNA damage induced by ionising radiation, and its depletion inhibits the efficiency of DSB repair by HR (Fang et al., 2015).

Cyclin F is a non-canonical cyclin which is a part of the SKP1-CUL1-F-box (SCF) E3 ubiquitin-protein ligase complex (Krajewski et al., 2020). Studies of skin cutaneous melanoma have suggested that cyclin F is involved in DNA repair (Gagat et al., 2018) and cell cycle progression (Yuan et al., 2019). Cyclin F interacts with ribonucleotide reductase family member 2 (RRM2), which catalyses the conversion of ribonucleotides to deoxyribonucleotides (dNTPs) necessary for DNA synthesis during replication and DNA repair. Defective elimination of cyclin F delays DNA repair and sensitises cells to DNA damage (D’Angiolella et al., 2012).

Senataxin functions in the resolution of R-loops. It plays important roles in maintaining genome integrity by co-ordinating transcription, DNA replication, and the DDR (Rawal et al., 2020). Yeast cells lacking functional *Sen1*, an ortholog of human senataxin, are characterised by the formation of DNA:RNA hybrids proximal to the break site, and defects in the fidelity of DNA repair and accurate processing of DSBs (Rawal et al., 2020). This is accompanied by prolonged binding of Ku70/80 at DSBs and increased mutagenic NHEJ events. Moreover, local DNA:RNA hybrids prime initiation of non-canonical Mre11- and Dna2-dependent DSB re-section, promoting HR repair (Rawal et al., 2020). At R-loop sites, senataxin also localises to sites of collision between components of the replisome and transcription apparatus, implying it functions at the interface of transcription and the DDR (Yeo et al., 2015). Loss of senataxin in human and mouse cells causes hypersensitivity to treatment with agents that induce either replication stress or R-loop formation, resulting in genomic instability and chromosomal rearrangement (Yeo et al., 2015).

Valosin-containing protein (VCP) is an AAA+ ATPase that is rapidly recruited to sites of DNA damage, where it removes K48-linked poly-ubiquitinated chromatin proteins and facilitates their turnover (Vaz et al., 2013). VCP is believed to be directly phosphorylated at Ser784 in response to DNA damage by phosphatidylinositol 3-kinase-related kinase (PIKK) family members with important functions in the DDR: ATM, ataxia- and Rad3-related (ATR), and DNA-PKcs (Blackford and Jackson, 2017). Inhibition of these kinases with caffeine or the more specific ATM inhibitor KU55933, perturbs VCP phosphorylation at Ser784 following etoposide treatment in HeLa cells (Zhu et al., 2020). Ser784 phosphorylation correlates with a decrease in VCP association with chromatin, cofactors NPL4/UFDI, and poly-ubiquitinated substrates (Zhu et al., 2020).

SQSTM1/p62 (sequestosome 1) selectively targets poly-ubiquitinated proteins for degradation via macro-autophagy and the proteasome (Hewitt et al., 2016). In the DDR, SQSTM1 facilitates proteasomal degradation of DNA repair machinery components filamin A (FLNA) and RAD51, resulting in slower repair of DSBs (Hewitt et al., 2016) and enhancement of NHEJ repair (Hewitt et al., 2016). In autophagy-defective cells, SQSTM1 inhibits ring finger protein 168 (RNF168), an E3 ligase, which is essential for the ubiquitination of histone H2A in response to DSBs (Wang et al., 2016). This event blocks the recruitment of DNA repair protein BRCA1, receptor-associated protein 80 (RAP80) and RAD51 to the sites of DSBs, resulting in impairment of DSB repair (Wang et al., 2016).

## Induction of Dna Damage in ALS by Pathological Forms of TDP-43, FUS, C9orf72, SOD 1, and NEK 1

Not surprisingly, DNA damage has been described following expression of mutant forms of the proteins described above in disease models. However, in addition, DNA damage is present following expression of other proteins associated with ALS, and has been detected in human patient tissues.

### TDP-43

Expression of ALS-associated TDP-43 mutants A315T (familial) and Q331K (sporadic) in neuronal NSC-34 cells results in impaired NHEJ compared to expression of wildtype TDP-43 (Konopka et al., 2020). Similarly, more DNA damage and less NHEJ was present in fibroblasts derived from ALS patients bearing the TDP-43 M337V mutation compared to fibroblasts from control individuals (Konopka et al., 2020). However, both TDP-43 mutants still co-immunoprecipitate and co-localise with 53BP1 foci, implying that they are recruited to sites of DNA damage, albeit to a lesser extent than wildtype TDP-43 (Konopka et al., 2020). Hence this implies that TDP-43 mutants possess direct deficits in DNA repair (Konopka et al., 2020). Expression of TDP-43 Q331K in SH-SY5Y cells results in less interaction of TDP-43 with the XRCC4-DNA ligase IV complex and enhanced cytosolic mis-localisation compared to wildtype TDP-43 (Guerrero et al., 2019). These abnormalities prevent the translocation of XRCC4-DNA ligase IV to the nucleus, which perturbs DNA repair (Guerrero et al., 2019). Human spinal cord tissue from a ALS TDP-43 Q331K patient displays accumulation of DSBs and more expression of γH2AX compared to age-matched controls (Guerrero et al., 2019). Therefore, together these data imply that pathological forms of TDP-43 in ALS impair DNA repair, leading to the accumulation of DNA damage and diminished genomic integrity. The presence of TDP-43 pathology in almost all ALS patients, including sporadic disease, also implies that in most ALS cases, depletion of TDP-43 from the nucleus inhibits DNA repair. Furthermore, it is known that the presence of unrepaired DNA lesions triggers apoptosis and leads to neurodegeneration (Stein and Toiber, 2017), suggesting that this is an important mechanism associated with pathophysiology in ALS.

DNA damage has also been shown to induce features of TDP-43 pathology – cytoplasmic mis-localisation and stress granule (SG) formation – in NSC-34 cells and mouse primary cortical neurons expressing either wildtype and or ALS-associated mutants A315T and Q331K (Konopka et al., 2020). Both wildtype and mutants are recruited to SGs, however, wildtype TDP-43 forms more SGs than the mutants (Konopka et al., 2020). Similarly, phosphorylated TDP-43-positive structures were also observed in the cytoplasm of TDP-43 M337V fibroblasts derived from pre-symptomatic ALS TDP-43 mutation carriers (Konopka et al., 2020). Moreover, pathological TDP-43 aggregates co-localise with SGs in ALS and FTD in human tissue and *in vitro* (Volkening et al., 2009; Liu-Yesucevitz et al., 2010). Thus, DNA damage triggered by TDP-43 pathology may contribute to neurodegeneration in the majority of ALS cases.

DNA damage was also detected in cortical neurons in early disease stages of a TDP-43 transgenic mouse model displaying cytoplasmic TDP-43 that lacks the nuclear localisation signal (NLS) (Konopka et al., 2020). Similarly, loss of nuclear TDP-43 strongly correlates with DSB repair defects, DDR activation and accumulation of DNA damage in a *Caenorhabditis elegans* strain with knockin of a *Tdp-1(ok803)* loss-of-function mutation, containing deletion of the C-terminal 299 residues containing the TDP-1 NLS and two TDP-1 RRMs (Mitra et al., 2019). In addition, conditional SH-SY5Y cell lines expressing wildtype or mutant TDP-43 Q331K displayed more cytosolic sequestration of poly-ubiquitinated, aggregated mutant TDP-43 than control lines (Guerrero et al., 2019). This correlated with increased genomic DNA strand breaks, and activation of phospho-ATM, phospho-53BP1, γH2AX and neuronal apoptosis (Guerrero et al., 2019).

Cytoplasmic aggregates immunoreactive for TDP-43 were identified in human sporadic ALS-derived fibroblasts (Riancho et al., 2020). These cells displayed more γH2AX and 53BP1 foci and were more vulnerable to induction of DNA damage by X-ray irradiation than control fibroblasts (Riancho et al., 2020). Mis-localisation of mutant TDP-43 A382T in neuronal SH-SY5Y cells and lymphoblastoid cell lines from an ALS patient resulted in the formation of R-loops, DSBs and Fanconi anemia repair centres, involved in the repair of the inter-strand crosslinks and replication fork blockages (Giannini et al., 2020). DNA damage has also been detected in neuronal genomes of sporadic ALS patients (Fitzmaurice et al., 1996; Ferrante et al., 1997; Kim B. et al., 2020).

Hence, in summary, the function of TDP-43 in DNA repair is perturbed in ALS and induction of DNA damage leads to the production of TDP-43 pathology and SGs. Both loss of TDP-43 functions in the nucleus as well as gain of toxic functions in the cytoplasm are implicated in neurodegeneration associated with the DDR in ALS.

### FUS

There is also considerable evidence for DNA damage associated with FUS in ALS. Motor neurons derived from FUS-P525L and P525L induced pluripotent stem cell (iPSC) lines show a significant delay in the repair of SSBs (Wang et al., 2018) following H_2_O_2_ treatment, implying there are deficits in oxidative DNA repair (Wang et al., 2018). Loss of function of FUS in the nucleus and defects in DNA nick ligation (Wang et al., 2018), due to less recruitment of XRCC1/LIG3 to SSBs (Wang et al., 2018), were observed. Similarly, α-motor neurons of lumbar spinal cords from FUS-ALS patients showed robust immunoreactivity to γH2AX in the nucleus (Naumann et al., 2018).

Analysis of genomic DNA integrity by long and accurate polymerase chain reaction (LA-PCR) detected ∼twofold DSBs or SSBs in ALS spinal cord tissue displaying cytosolic accumulation of FUS relative to control tissue (Wang et al., 2018). In another study involving human iPSCs-derived motor neurons, mutation of the FUS NLS resulted in impairment of PARP-1 dependent DDR signalling, leading to DSB formation, neurodegeneration and the formation of FUS aggregates (Naumann et al., 2018), implying that DNA damage is an early event in the pathophysiology of FUS-ALS (Naumann et al., 2018). In addition, etoposide treatment of these cells leads to FUS mis-localisation to the cytoplasm and inclusion formation, implying that DNA damage is upstream to the formation of pathological forms of FUS (Naumann et al., 2018). FUS-GFP was recruited rapidly to DNA damage sites in wildtype IPSC-derived motor neurons unlike FUS-NLS mutant lines (Naumann et al., 2018). This could be rescued by restoration of nuclear import of FUS by inhibition of arginine methylation using adenosine-2,3-dialdehyde (AdOx) (Naumann et al., 2018) or prevention of nuclear export of FUS by inhibition of DNA-PK with inhibitor NU7442 (Naumann et al., 2018). Together these studies imply the existence of a link between the pathological location of FUS and DNA damage. Thus, in summary, similar to TDP-43, in FUS-associated ALS, impaired DDR signalling may lead to neurodegeneration and aggregate formation. These studies indicate the need for novel therapeutic approaches aimed at restoring DNA repair in ALS, particularly those that are upstream to aggregate formation.

### C9orf72

Several lines of evidence demonstrate that DNA damage is induced by C9orf72 hexanucleotide repeat expansions in ALS/FTD. This results in the formation of both R loops and G quadruplexes, inducing nucleolar stress and DNA damage (Farg et al., 2017; Walker et al., 2017), which has been detected in both C9orf72-ALS patient tissues and neuronal cells (Farg et al., 2017). C9orf72 hexanucleotide repeat expansions induce the formation of greatly expanded RNA which form foci that sequester DNA repair proteins, including TDP-43, FUS, NPM1 and APE1, which would therefore disrupt their normal DNA repair functions (Lee et al., 2013; Cooper-Knock et al., 2014). DPRs induce the formation of DSBs, which are further enhanced following knockout of heterogeneous ribonucleoprotein hnRNPA3 (Nihei et al., 2020). In the dentate gyrus of C9orf72 patient brains, lower nuclear hnRNPA3 levels were associated with increased DNA damage (Nihei et al., 2020). Expression of the DPRs polyGA and polyPR promote the formation of phosphorylated ATM foci, a major sensor of DSBs, whereas polyGA reduces formation of phosphorylated ATM foci (Nihei et al., 2020). Furthermore, the presence of DPR polyGA increases oxidative stress and DNA damage in iPSC-derived motor neurons. This was associated with binding to mitochondrial ribosomal proteins, compromising mitochondrial functions (Lopez-Gonzalez et al., 2016). Another possible mechanism by which the DPRs can perturb DNA repair is related to the nuclear envelope. C9orf72 DPRs are known to inhibit nucleocytoplasmic transport and disrupt the nuclear pore complex (Freibaum et al., 2015; Jovicic et al., 2015). More recently, defects in the architecture of the nuclear envelope were linked to the impairment of genome integrity and induction of DNA damage (Cancer Discovery, 2021; Rodriguez-Munoz et al., 2022). However, the direct relationship between DPRs, DNA damage and nuclear envelop in ALS requires further investigation.

### SOD 1

ALS-associated mutations in SOD1 have also been linked to DNA damage. More DNA damage and apoptosis induced by activation of p53 were observed in SH-SY5Y cells expressing mutant SOD1 G93A compared to cells overexpressing wildtype SOD1 (Barbosa et al., 2010). Similarly, over-expression of SOD1 G93A in NSC-34 cells activates the DDR and downregulates expression of human SpeedyA1 (Spy1), which is responsible for cellular survival and inhibition of DNA damage-induced apoptosis (Wang et al., 2019). However, in contrast, in human iPSC-derived motor neurons bearing SOD1 mutations G93A or A4V, the DDR and DNA repair were found to be unchanged compared to isogenic controls, implying that SOD1 mutations have no effect on DNA damage (Kim B. et al., 2020). SOD1 catalyses the conversion of superoxide anions into hydrogen peroxide, thus protecting the cell from oxidative damage (Fridovich, 1997). Interestingly, in response to elevated endogenous or exogenous reactive oxygen species (ROS), SOD1 relocates to the nucleus, where it regulates expression of oxidative resistance and DNA repair genes (Tsang et al., 2014). In ALS, the cytoplasmic accumulation of mutant SOD1 G93A has been linked to both impairment of the protective activity of SOD1 against oxidative stress, as well as disruption of expression of these protective genes, leading to the accumulation of DNA damage (Sau et al., 2007).

### NEK 1

NEK1 was only recently associated with ALS, where genetic risk variants were detected in ∼3% of European and European-American ALS cases (Kenna et al., 2016). To date there has been only one publication related to the DNA repair function of NEK1 in ALS. Mutant NEK1c.2434A>T hiPSC-derived motor neurons displayed significantly more DNA damage and impaired DDR compared to wildtype neurons, both in the absence of DNA damage inducing agents and following DNA damage induced by γ-irradiation, in a maturation-dependent manner (Higelin et al., 2018). Given the role of NEK1 in the cellular response to genotoxic stress (Pelegrini et al., 2010) and the lack of knowledge about its role in ALS, future investigations into the link between the function of NEK1 in DNA repair and ALS may bring new insights into its role in pathogenesis.

## Conclusion

**FIGURE 3:**
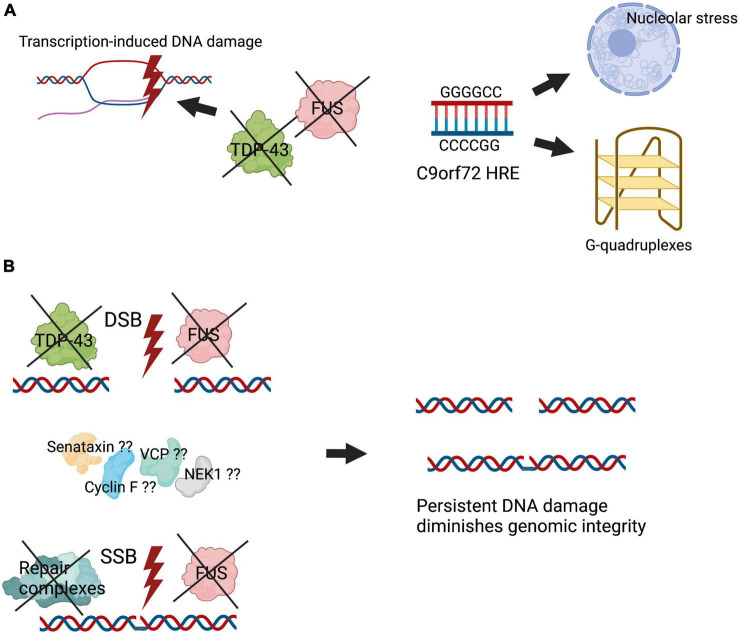
Illustration summarising how DNA damage induces neurodegeneration in ALS. Genomic integrity is essential for maintenance of cellular homeostasis. However, defective DNA repair processes diminish genomic integrity, leading to mutations and cell death. **(A)** The presence of specific mutations in ALS, including the C9orf72 hexanucleotide repeat expansion, induces the formation of aberrant DNA structures such as R loops, inducing stress in the nucleolus. In addition, key DNA repair proteins, including TDP-43 and FUS, are dysfunctional and form aberrant pathological forms in ALS, causing transcription-induced DNA damage. **(B)** Inefficient binding of TDP-43 and FUS at sites of DNA damage, as well as impairment of their activities in DNA repair in their pathological forms, inhibit the assembly or stabilisation of key DNA repair complexes. Putatively, other proteins associated with ALS, including mutant forms of senataxin, VCP, cyclin F, and NEK1, may also lead to deficiencies in DNA repair. These events lead to accumulation of DNA damage and impaired genomic integrity, which triggers neurodegeneration.

Accumulation of DNA damage is one of the major cellular insults involved in aging, which is the most important risk factor for neurodegeneration. Increasing evidence implicates a role for DNA damage and impaired DNA repair processes in ALS. Early studies described increased oxidative DNA damage in ALS tissues and models. However, these initial findings have now been significantly expanded, and they suggest that DNA damage is a central pathogenic mechanism in ALS. DNA damage has been observed in disease models involving multiple different proteins associated with both sporadic and familial forms of ALS. Increasingly, proteins with roles in DNA repair are found to be mutated in ALS, implicating loss of these normal cellular functions in pathophysiology. However, DNA damage is detected in ALS in the absence of proteins with a known function in DNA repair, suggesting that future studies may reveal more ALS proteins engaged in DNA repair. DNA damage has also been detected early in disease course in animal models, implying that it is directly involved in pathophysiology. However, it is unclear whether DNA damage is a direct cause of neurodegeneration in ALS or whether it results from other disease processes. Nevertheless, the direct involvement of TDP-43 and FUS in DNA repair mechanisms is significant given that TDP-43 is implicated in a pathological form in 97% of ALS cases (Heyburn and Moussa, 2017) and similarly, FUS pathology is now thought to be present in sporadic ALS (90% of all ALS) (Tyzack et al., 2019). Together these findings imply that DNA damage and defective DNA repair are a common and central feature in ALS pathogenesis. Moreover, they highlight the importance of future studies aimed at inhibiting DNA damage or restoring DNA repair as novel therapeutic strategies for ALS. Figure 3 summarises our current understanding of how DNA damage induces neurodegeneration in ALS. Given the link between TDP-43 pathology and DNA damage, it is possible that therapies that target the DDR may be widely applicable in ALS, such as PARP-1 inhibitors, which are known to inhibit neuronal death. However these approaches have not been evaluated clinically (Thapa et al., 2021).

## Author Contributions

AK conceptualized the manuscript. JA provided resources. Both AK and JA wrote the manuscript and edited it for publication.

## Conflict of Interest

The authors declare that the research was conducted in the absence of any commercial or financial relationships that could be construed as a potential conflict of interest.

## Publisher’s Note

All claims expressed in this article are solely those of the authors and do not necessarily represent those of their affiliated organizations, or those of the publisher, the editors and the reviewers. Any product that may be evaluated in this article, or claim that may be made by its manufacturer, is not guaranteed or endorsed by the publisher.

## Footnotes

1
https://www.nature.com/collections/hwnqqcstyj/

## References

[B1] Abramzon Y. A. Fratta P. Traynor B. J. Chia R. (2020). The overlapping genetics of amyotrophic lateral sclerosis and frontotemporal dementia. *Front. Neurosci.* 14:42. 10.3389/fnins.2020.00042 32116499 PMC7012787

[B2] Agathangelou K. Apostolou Z. Garinis G. A. (2018). Nuclear DNA damage and ageing. *Subcell. Biochem.* 90 309–322. 30779013 10.1007/978-981-13-2835-0_10

[B3] Ahnesorg P. Smith P. Jackson S. P. (2006). XLF interacts with the XRCC4-DNA ligase IV complex to promote DNA nonhomologous end-joining. *Cell* 124 301–313. 10.1016/j.cell.2005.12.031 16439205

[B4] Al Khleifat A. Iacoangeli A. van Vugt J. Bowles H. Moisse M. Zwamborn R. A. J. (2022). Structural variation analysis of 6,500 whole genome sequences in amyotrophic lateral sclerosis. *NPJ Genom. Med.* 7:8. 10.1038/s41525-021-00267-9 35091648 PMC8799638

[B5] Al-Saif A. Al-Mohanna F. Bohlega S. (2011). A mutation in sigma-1 receptor causes juvenile amyotrophic lateral sclerosis. *Ann Neurol.* 70 913–919. 10.1002/ana.22534 21842496

[B6] Altmeyer M. Neelsen K. J. Teloni F. Pozdnyakova I. Pellegrino S. Grofte M. (2015). Liquid demixing of intrinsically disordered proteins is seeded by poly(ADP-ribose). *Nat Commun.* 6:8088. 10.1038/ncomms9088 26286827 PMC4560800

[B7] Balendra R. Isaacs A. M. (2018). C9orf72-mediated ALS and FTD: multiple pathways to disease. *Nat. Rev. Neurol.* 14 544–558. 10.1038/s41582-018-0047-2 30120348 PMC6417666

[B8] Barbosa L. F. Cerqueira F. M. Macedo A. F. Garcia C. C. Angeli J. P. Schumacher R. I. (2010). Increased SOD1 association with chromatin, DNA damage, p53 activation, and apoptosis in a cellular model of SOD1-linked ALS. *Biochim. Biophys. Acta* 1802 462–471. 10.1016/j.bbadis.2010.01.011 20097285

[B9] Barroso S. Herrera-Moyano E. Munoz S. Garcia-Rubio M. Gomez-Gonzalez B. Aguilera A. (2019). The DNA damage response acts as a safeguard against harmful DNA-RNA hybrids of different origins. *EMBO Rep.* 20:e47250. 10.15252/embr.201847250 31338941 PMC6726908

[B10] Bennett C. L. Dastidar S. G. Ling S. C. Malik B. Ashe T. Wadhwa M. (2018). Senataxin mutations elicit motor neuron degeneration phenotypes and yield TDP-43 mislocalization in ALS4 mice and human patients. *Acta Neuropathol.* 136 425–443. 10.1007/s00401-018-1852-9 29725819 PMC6098723

[B11] Blackford A. N. Jackson S. P. A. T. M. (2017). ATR, and DNA-PK: the trinity at the heart of the DNA damage response. *Mol Cell.* 66 801–817. 10.1016/j.molcel.2017.05.015 28622525

[B12] Brandsma I. Gent D. C. (2012). Pathway choice in DNA double strand break repair: observations of a balancing act. *Genome Integr.* 3:9. 10.1186/2041-9414-3-9 23181949 PMC3557175

[B13] Brenner D. Muller K. Wieland T. Weydt P. Bohm S. Lule D. (2016). NEK1 mutations in familial amyotrophic lateral sclerosis. *Brain* 139(Pt 5):e28. 10.1093/brain/aww033 26945885

[B14] Brettle M. Stefen H. Djordjevic A. Fok S. Y. Y. Chan J. W. van Hummel A. (2019). Developmental expression of mutant PFN1 in motor neurons impacts neuronal growth and motor performance of young and adult mice. *Front. Mol. Neurosci.* 12:231. 10.3389/fnmol.2019.00231 31611772 PMC6776973

[B15] Buck D. Malivert L. de Chasseval R. Barraud A. Fondaneche M. C. Sanal O. (2006). Cernunnos, a novel nonhomologous end-joining factor, is mutated in human immunodeficiency with microcephaly. *Cell* 124 287–299. 10.1016/j.cell.2005.12.030 16439204

[B16] Burge S. Parkinson G. N. Hazel P. Todd A. K. Neidle S. (2006). Quadruplex DNA: sequence, topology and structure. *Nucleic Acids Res.* 34 5402–5415. 10.1093/nar/gkl655 17012276 PMC1636468

[B17] Cancer Discovery (2021). Nuclear envelope disruptions induce DNA damage and invasiveness. *Cancer Discov.* 11:OF5. 10.1158/2159-8290.CD-RW2021-138 34598943

[B18] Carusillo A. Mussolino C. D. N. A. (2020). Damage: from threat to treatment. *Cells* 9:1665. 10.3390/cells9071665 32664329 PMC7408370

[B19] Chatterjee N. Walker G. C. (2017). Mechanisms of DNA damage, repair, and mutagenesis. *Environ. Mol. Mutagen.* 58 235–263. 28485537 10.1002/em.22087 PMC5474181

[B20] Chen Y. Z. Hashemi S. H. Anderson S. K. Huang Y. Moreira M. C. Lynch D. R. (2006). Senataxin, the yeast Sen1p orthologue: characterization of a unique protein in which recessive mutations cause ataxia and dominant mutations cause motor neuron disease. *Neurobiol. Dis.* 23 97–108. 10.1016/j.nbd.2006.02.007 16644229

[B21] Chia R. Chio A. Traynor B. J. (2018). Novel genes associated with amyotrophic lateral sclerosis: diagnostic and clinical implications. *Lancet Neurol.* 17 94–102. 10.1016/S1474-4422(17)30401-5 29154141 PMC5901717

[B22] Ciccia A. Elledge S. J. (2010). The DNA damage response: making it safe to play with knives. *Mol. Cell* 40 179–204. 10.1016/j.molcel.2010.09.019 20965415 PMC2988877

[B23] Cirulli E. T. Lasseigne B. N. Petrovski S. Sapp P. C. Dion P. A. Leblond C. S. (2015). Exome sequencing in amyotrophic lateral sclerosis identifies risk genes and pathways. *Science* 347 1436–1441. 10.1126/science.aaa3650 25700176 PMC4437632

[B24] Clark C. M. Forman M. S. (2006). Frontotemporal lobar degeneration with motor neuron disease: a clinical and pathological spectrum. *Arch. Neurol.* 63 489–490. 10.1001/archneur.63.4.489 16606759

[B25] Cooper-Knock J. Walsh M. J. Higginbottom A. Robin Highley J. Dickman M. J. Edbauer D. (2014). Sequestration of multiple RNA recognition motif-containing proteins by C9orf72 repeat expansions. *Brain.* 137(Pt 7) 2040–2051. 10.1093/brain/awu120 24866055 PMC4065024

[B26] Couthouis J. Raphael A. R. Daneshjou R. Gitler A. D. (2014). Targeted exon capture and sequencing in sporadic amyotrophic lateral sclerosis. *PLoS Genet.* 10:e1004704. 10.1371/journal.pgen.1004704 25299611 PMC4191946

[B27] D’Angiolella V. Donato V. Forrester F. M. Jeong Y. T. Pellacani C. Kudo Y. (2012). Cyclin F-mediated degradation of ribonucleotide reductase M2 controls genome integrity and DNA repair. *Cell* 149 1023–1034. 10.1016/j.cell.2012.03.043 22632967 PMC3616325

[B28] Davidovic L. Vodenicharov M. Affar E. B. Poirier G. G. (2001). Importance of poly(ADP-ribose) glycohydrolase in the control of poly(ADP-ribose) metabolism. *Exp. Cell Res.* 268 7–13. 10.1006/excr.2001.5263 11461113

[B29] DeJesus-Hernandez M. Mackenzie I. R. Boeve B. F. Boxer A. L. Baker M. Rutherford N. J. (2011). Expanded GGGGCC hexanucleotide repeat in noncoding region of C9ORF72 causes chromosome 9p-linked FTD and ALS. *Neuron* 72 245–256. 10.1016/j.neuron.2011.09.011 21944778 PMC3202986

[B30] Deng H. X. Chen W. Hong S. T. Boycott K. M. Gorrie G. H. Siddique N. (2011). Mutations in UBQLN2 cause dominant X-linked juvenile and adult-onset ALS and ALS/dementia. *Nature* 477 211–215. 10.1038/nature10353 21857683 PMC3169705

[B31] Deng H. Gao K. Jankovic J. (2014). The role of FUS gene variants in neurodegenerative diseases. *Nat. Rev. Neurol.* 10 337–348. 10.1038/nrneurol.2014.78 24840975

[B32] DNA Damage Repair (2021). *DNA Damage Repair [Internet].* Available online at: https://www.nature.com/collections/hwnqqcstyj/ (accessed November 2, 2021).

[B33] Fang F. Al-Chalabi A. Ronnevi L. O. Turner M. R. Wirdefeldt K. Kamel F. (2013). Amyotrophic lateral sclerosis and cancer: a register-based study in Sweden. *Amyotroph. Lateral Scler. Frontotemporal. Degener.* 14 362–368. 10.3109/21678421.2013.775309 23527497 PMC5451142

[B34] Fang X. Lin H. Wang X. Zuo Q. Qin J. Zhang P. (2015). The NEK1 interactor, C21ORF2, is required for efficient DNA damage repair. *Acta Biochim. Biophys. Sin. (Shanghai)* 47 834–841. 10.1093/abbs/gmv076 26290490 PMC4581587

[B35] Farg M. A. Konopka A. Soo K. Y. Ito D. Atkin J. D. (2017). The DNA damage response (DDR) is induced by the C9orf72 repeat expansion in amyotrophic lateral sclerosis. *Hum. Mol. Genet.* 26 2882–2896. 10.1093/hmg/ddx170 28481984

[B36] Fecto F. Siddique T. (2011). Making connections: pathology and genetics link amyotrophic lateral sclerosis with frontotemporal lobe dementia. *J. Mol. Neurosci.* 45 663–675. 10.1007/s12031-011-9637-9 21901496

[B37] Fecto F. Yan J. Vemula S. P. Liu E. Yang Y. Chen W. (2011). SQSTM1 mutations in familial and sporadic amyotrophic lateral sclerosis. *Arch. Neurol.* 68 1440–1446. 10.1001/archneurol.2011.250 22084127

[B38] Ferrante R. J. Browne S. E. Shinobu L. A. Bowling A. C. Baik M. J. MacGarvey U. (1997). Evidence of increased oxidative damage in both sporadic and familial amyotrophic lateral sclerosis. *J. Neurochem.* 69 2064–2074. 10.1046/j.1471-4159.1997.69052064.x 9349552

[B39] Fitzmaurice P. S. Shaw I. C. Kleiner H. E. Miller R. T. Monks T. J. Lau S. S. (1996). Evidence for DNA damage in amyotrophic lateral sclerosis. *Muscle Nerve* 19 797–798. 8609941

[B40] Fousteri M. Vermeulen W. van Zeeland A. A. Mullenders L. H. (2006). Cockayne syndrome A and B proteins differentially regulate recruitment of chromatin remodeling and repair factors to stalled RNA polymerase II in vivo. *Mol. Cell* 23 471–482. 16916636 10.1016/j.molcel.2006.06.029

[B41] Freedman D. M. Curtis R. E. Daugherty S. E. Goedert J. J. Kuncl R. W. Tucker M. A. (2013). The association between cancer and amyotrophic lateral sclerosis. *Cancer Causes Control* 24 55–60. 23090035 10.1007/s10552-012-0089-5 PMC3529829

[B42] Freedman D. M. Travis L. B. Gridley G. Kuncl R. W. (2005). Amyotrophic lateral sclerosis mortality in 1.9 million US cancer survivors. *Neuroepidemiology* 25 176–180. 10.1159/000087447 16103728

[B43] Freibaum B. D. Taylor J. P. (2017). The role of dipeptide repeats in C9ORF72-Related ALS-FTD. *Front. Mol. Neurosci.* 10:35. 10.3389/fnmol.2017.00035 28243191 PMC5303742

[B44] Freibaum B. D. Chitta R. K. High A. A. Taylor J. P. (2010). Global analysis of TDP-43 interacting proteins reveals strong association with RNA splicing and translation machinery. *J. Proteome Res.* 9 1104–1120. 10.1021/pr901076y 20020773 PMC2897173

[B45] Freibaum B. D. Lu Y. Lopez-Gonzalez R. Kim N. C. Almeida S. Lee K. H. (2015). GGGGCC repeat expansion in C9orf72 compromises nucleocytoplasmic transport. *Nature* 525 129–133. 10.1038/nature14974 26308899 PMC4631399

[B46] Fridovich I. (1997). Superoxide anion radical (O2-.), superoxide dismutases, and related matters. *J. Biol. Chem.* 272 18515–18517. 10.1074/jbc.272.30.18515 9228011

[B47] Gagat M. Krajewski A. Grzanka D. Grzanka A. (2018). Potential role of cyclin F mRNA expression in the survival of skin melanoma patients: comprehensive analysis of the pathways altered due to cyclin F upregulation. *Oncol. Rep.* 40 123–144. 10.3892/or.2018.6435 29767233 PMC6059736

[B48] Gao F. B. Almeida S. Lopez-Gonzalez R. (2017). Dysregulated molecular pathways in amyotrophic lateral sclerosis–frontotemporal dementia spectrum disorder. *EMBO J.* 36 2931–2950. 10.15252/embj.201797568 28916614 PMC5641681

[B49] Garcia-Muse T. Aguilera A. R. (2019). Loops: from physiological to pathological roles. *Cell* 179 604–618. 10.1016/j.cell.2019.08.055 31607512

[B50] Gendron T. F. Petrucelli L. (2018). Disease mechanisms of C9ORF72 repeat expansions. *Cold Spring Harb. Perspect. Med.* 8:a024224. 10.1101/cshperspect.a024224 28130314 PMC5880161

[B51] Giannini M. Bayona-Feliu A. Sproviero D. Barroso S. I. Cereda C. Aguilera A. (2020). TDP-43 mutations link amyotrophic lateral sclerosis with R-loop homeostasis and R loop-mediated DNA damage. *PLoS Genet.* 16:e1009260. 10.1371/journal.pgen.1009260 33301444 PMC7755276

[B52] Gilley J. Jackson O. Pipis M. Estiar M. A. Al-Chalabi A. Danzi M. C. (2021). Enrichment of SARM1 alleles encoding variants with constitutively hyperactive NADase in patients with ALS and other motor nerve disorders. *Elife* 10:e70905. 10.7554/eLife.70905 34796871 PMC8735862

[B53] Guerrero E. N. Mitra J. Wang H. Rangaswamy S. Hegde P. M. Basu P. (2019). Amyotrophic lateral sclerosis-associated TDP-43 mutation Q331K prevents nuclear translocation of XRCC4-DNA ligase 4 complex and is linked to genome damage-mediated neuronal apoptosis. *Hum. Mol. Genet.* 28 3161–3162. 31504503 10.1093/hmg/ddz141 PMC6867871

[B54] Hand C. K. Devon R. S. Gros-Louis F. Rochefort D. Khoris J. Meininger V. (2003). Mutation screening of the ALS2 gene in sporadic and familial amyotrophic lateral sclerosis. *Arch. Neurol.* 60 1768–1771. 10.1001/archneur.60.12.1768 14676054

[B55] Harley J. Hagemann C. Serio A. Patani R. (2020). FUS is lost from nuclei and gained in neurites of motor neurons in a human stem cell model of VCP-related ALS. *Brain* 143:e103. 10.1093/brain/awaa339 33253377 PMC7805784

[B56] Harper J. W. Elledge S. J. (2007). The DNA damage response: ten years after. *Mol. Cell* 28 739–745. 10.1016/j.molcel.2007.11.015 18082599

[B57] Hewitt G. Carroll B. Sarallah R. Correia-Melo C. Ogrodnik M. Nelson G. (2016). SQSTM1/p62 mediates crosstalk between autophagy and the UPS in DNA repair. *Autophagy* 12 1917–1930. 10.1080/15548627.2016.1210368 27391408 PMC5391493

[B58] Heyburn L. Moussa C. E. (2017). TDP-43 in the spectrum of MND-FTLD pathologies. *Mol. Cell Neurosci.* 83 46–54. 10.1016/j.mcn.2017.07.001 28687523 PMC5581706

[B59] Higelin J. Catanese A. Semelink-Sedlacek L. L. Oeztuerk S. Lutz A. K. Bausinger J. (2018). NEK1 loss-of-function mutation induces DNA damage accumulation in ALS patient-derived motoneurons. *Stem Cell Res.* 30 150–162. 10.1016/j.scr.2018.06.005 29929116

[B60] Hill S. J. Mordes D. A. Cameron L. A. Neuberg D. S. Landini S. Eggan K. (2016). Two familial ALS proteins function in prevention/repair of transcription-associated DNA damage. *Proc. Natl. Acad. Sci. U.S.A.* 113 E7701–E7709. 10.1073/pnas.1611673113 27849576 PMC5137757

[B61] Huang X. Shen S. Fan D. (2017). No evidence for pathogenic role of UBQLN2 mutations in sporadic amyotrophic lateral sclerosis in the mainland chinese population. *PLoS One* 12:e0170943. eCollection 2017 10.1371/journal.pone.0170943 28125704 PMC5268382

[B62] Ikenaka K. Ishigaki S. Iguchi Y. Kawai K. Fujioka Y. Yokoi S. (2020). Characteristic features of FUS inclusions in spinal motor neurons of sporadic amyotrophic lateral sclerosis. *J. Neuropathol. Exp. Neurol.* 79 370–377. 10.1093/jnen/nlaa003 32142134

[B63] Iyama T. Wilson D. M. III (2013). DNA repair mechanisms in dividing and non-dividing cells. *DNA Repair. (Amst)* 12 620–636. 10.1016/j.dnarep.2013.04.015 23684800 PMC3720834

[B64] Jackson S. P. Bartek J. (2009). The DNA-damage response in human biology and disease. *Nature* 461 1071–1078. 10.1038/nature08467 19847258 PMC2906700

[B65] Johnson J. O. Mandrioli J. Benatar M. Abramzon Y. Van Deerlin V. M. Trojanowski J. Q. (2010). Exome sequencing reveals VCP mutations as a cause of familial ALS. *Neuron* 68 857–864. 10.1016/j.neuron.2010.11.036 21145000 PMC3032425

[B66] Jovicic A. Mertens J. Boeynaems S. Bogaert E. Chai N. Yamada S. B. (2015). Modifiers of C9orf72 dipeptide repeat toxicity connect nucleocytoplasmic transport defects to FTD/ALS. *Nat. Neurosci.* 18 1226–1229. 10.1038/nn.4085 26308983 PMC4552077

[B67] Kaneb H. M. Folkmann A. W. Belzil V. V. Jao L. E. Leblond C. S. Girard S. L. (2015). Deleterious mutations in the essential mRNA metabolism factor, hGle1, in amyotrophic lateral sclerosis. *Hum. Mol. Genet.* 24 1363–1373. 10.1093/hmg/ddu545 25343993 PMC4321443

[B68] Kawaguchi T. Rollins M. G. Moinpour M. Morera A. A. Ebmeier C. C. Old W. M. (2020). Changes to the TDP-43 and FUS interactomes induced by DNA damage. *J. Proteome Res.* 19 360–370. 10.1021/acs.jproteome.9b00575 31693373 PMC6947635

[B69] Kenna K. P. McLaughlin R. L. Byrne S. Elamin M. Heverin M. Kenny E. M. (2013). Delineating the genetic heterogeneity of ALS using targeted high-throughput sequencing. *J. Med. Genet.* 50 776–783. 10.1136/jmedgenet-2013-101795 23881933 PMC3812897

[B70] Kenna K. P. van Doormaal P. T. Dekker A. M. Ticozzi N. Kenna B. J. Diekstra F. P. (2016). NEK1 variants confer susceptibility to amyotrophic lateral sclerosis. *Nat. Genet.* 48 1037–1042. 10.1038/ng.3626 27455347 PMC5560030

[B71] Kim B. Jeong Y. E. Wong M. Martin L. J. (2020). DNA damage accumulates and responses are engaged in human ALS brain and spinal motor neurons and DNA repair is activatable in iPSC-derived motor neurons with SOD1 mutations. *Acta Neuropathol. Commun.* 8:7. 10.1186/s40478-019-0874-4 32005289 PMC6995159

[B72] Kim G. Gautier O. Tassoni-Tsuchida E. Ma X. R. Gitler A. D. (2020). ALS genetics: gains, losses, and implications for future therapies. *Neuron* 108 822–842. 10.1016/j.neuron.2020.08.022 32931756 PMC7736125

[B73] Kim H. J. Kim N. C. Wang Y. D. Scarborough E. A. Moore J. Diaz Z. (2013). Mutations in prion-like domains in hnRNPA2B1 and hnRNPA1 cause multisystem proteinopathy and ALS. *Nature* 495 467–473. 10.1038/nature11922 23455423 PMC3756911

[B74] Kok J. R. Palminha N. M. Dos Santos Souza C. El-Khamisy S. F. Ferraiuolo L. (2021). DNA damage as a mechanism of neurodegeneration in ALS and a contributor to astrocyte toxicity. *Cell Mol. Life Sci.* 78 5707–5729. 10.1007/s00018-021-03872-0 34173837 PMC8316199

[B75] Konopka A. Whelan D. R. Jamali M. S. Perri E. Shahheydari H. Toth R. P. (2020). Impaired NHEJ repair in amyotrophic lateral sclerosis is associated with TDP-43 mutations. *Mol. Neurodegener.* 15:51. 10.1186/s13024-020-00386-4 32907630 PMC7488163

[B76] Koppers M. van Blitterswijk M. M. Vlam L. Rowicka P. A. van Vught P. W. Groen E. J. (2012). VCP mutations in familial and sporadic amyotrophic lateral sclerosis. *Neurobiol. Aging* 33 837.e7–e13. 10.1016/j.neurobiolaging.2011.10.006 22078486

[B77] Krajewski A. Gagat M. Zuryn A. Halas-Wisniewska M. Grzanka D. Grzanka A. (2020). Cyclin F is involved in response to cisplatin treatment in melanoma cell lines. *Oncol. Rep.* 43 765–772. 10.3892/or.2020.7465 32020229 PMC7040885

[B78] Krokan H. E. Bjoras M. (2013). Base excision repair. *Cold Spring Harb. Perspect. Biol.* 5:a012583. 10.1101/cshperspect.a012583 PMC3683898 23545420

[B79] Lai C. K. Gupta N. Wen X. Rangell L. Chih B. Peterson A. S. (2011). Functional characterization of putative cilia genes by high-content analysis. *Mol. Biol. Cell.* 22 1104–1119. 10.1091/mbc.E10-07-0596 21289087 PMC3069013

[B80] Lans H. Hoeijmakers J. H. J. Vermeulen W. Marteijn J. A. (2019). The DNA damage response to transcription stress. *Nat. Rev. Mol. Cell Biol.* 20 766–784. 31558824 10.1038/s41580-019-0169-4

[B81] Larry H. T. Charles L. L. (2000–2013). *Origin, Recognition, Signaling and Repair of DNA Double-Strand Breaks in Mammalian Cells.* Austin, TX: Landes Bioscience.

[B82] Lee E. B. Lee V. M. Trojanowski J. Q. (2011). Gains or losses: molecular mechanisms of TDP43-mediated neurodegeneration. *Nat. Rev. Neurosci.* 13 38–50. 10.1038/nrn3121 22127299 PMC3285250

[B83] Lee J. H. Paull T. T. (2021). Cellular functions of the protein kinase ATM and their relevance to human disease. *Nat. Rev. Mol. Cell Biol.* 22 796–814. 10.1038/s41580-021-00394-2 34429537

[B84] Lee Y. B. Chen H. J. Peres J. N. Gomez-Deza J. Attig J. Stalekar M. (2013). Hexanucleotide repeats in ALS/FTD form length-dependent RNA foci, sequester RNA binding proteins, and are neurotoxic. *Cell Rep.* 5 1178–1186. 10.1016/j.celrep.2013.10.049 24290757 PMC3898469

[B85] Li G. M. (2008). Mechanisms and functions of DNA mismatch repair. *Cell Res.* 18 85–98. 18157157 10.1038/cr.2007.115

[B86] Li S. Shi B. Liu X. An H. X. (2020). Acetylation and deacetylation of DNA repair proteins in cancers. *Front. Oncol.* 10:573502. 10.3389/fonc.2020.573502 33194676 PMC7642810

[B87] Lieber M. R. (2008). The mechanism of human nonhomologous DNA end joining. *J. Biol. Chem.* 283 1–5. 17999957 10.1074/jbc.R700039200

[B88] Lieber M. R. (2010). The mechanism of double-strand DNA break repair by the nonhomologous DNA end-joining pathway. *Annu. Rev. Biochem.* 79 181–211. 10.1146/annurev.biochem.052308.093131 20192759 PMC3079308

[B89] Lindahl T. Barnes D. E. (2000). Repair of endogenous DNA damage. *Cold Spring Harb. Symp. Quant. Biol.* 65 127–133. 12760027 10.1101/sqb.2000.65.127

[B90] Ling S.-C. Polymenidou M. Cleveland D. W. (2013). Converging mechanisms in ALS and FTD: disrupted RNA and protein homeostasis. *Neuron* 79 416–438. 10.1016/j.neuron.2013.07.033 23931993 PMC4411085

[B91] Liu-Yesucevitz L. Bilgutay A. Zhang Y. J. Vanderweyde T. Citro A. Mehta T. (2010). Tar DNA binding protein-43 (TDP-43) associates with stress granules: analysis of cultured cells and pathological brain tissue. *PLoS One* 5:e13250. 10.1371/journal.pone.0013250 20948999 PMC2952586

[B92] Lomen-Hoerth C. Anderson T. Miller B. (2002). The overlap of amyotrophic lateral sclerosis and frontotemporal dementia. *Neurology* 59 1077–1079. 12370467 10.1212/wnl.59.7.1077

[B93] Lopez-Gonzalez R. Lu Y. Gendron T. F. Karydas A. Tran H. Yang D. (2016). Poly(GR) in C9ORF72-related ALS/FTD compromises mitochondrial function and increases oxidative stress and DNA damage in iPSC-derived motor neurons. *Neuron* 92 383–391. 10.1016/j.neuron.2016.09.015 27720481 PMC5111366

[B94] Ma A. Dai X. (2018). The relationship between DNA single-stranded damage response and double-stranded damage response. *Cell Cycle* 17 73–79. 10.1080/15384101.2017.1403681 29157089 PMC5815444

[B95] Mackenzie I. R. Nicholson A. M. Sarkar M. Messing J. Purice M. D. Pottier C. (2017). TIA1 mutations in amyotrophic lateral sclerosis and frontotemporal dementia promote phase separation and alter stress granule dynamics. *Neuron* 95 808–816 e9. 10.1016/j.neuron.2017.07.025 28817800 PMC5576574

[B96] Mandrioli J. Mediani L. Alberti S. Carra S. (eds) (2020). “ALS and FTD: Where RNA metabolism meets protein quality control,” in *Seminars in Cell & Developmental Biology*, (Amsterdam: Elsevier). 10.1016/j.semcdb.2019.06.003 31254610

[B97] Mastrocola A. S. Kim S. H. Trinh A. T. Rodenkirch L. A. Tibbetts R. S. (2013). The RNA-binding protein fused in sarcoma (FUS) functions downstream of poly(ADP-ribose) polymerase (PARP) in response to DNA damage. *J. Biol. Chem.* 288 24731–24741. 10.1074/jbc.M113.497974 23833192 PMC3750169

[B98] McCann E. P. Henden L. Fifita J. A. Zhang K. Y. Grima N. Bauer D. C. (2021). Evidence for polygenic and oligogenic basis of Australian sporadic amyotrophic lateral sclerosis. *J. Med. Genet.* 58, 87–95. 10.1136/jmedgenet-2020-106866 32409511

[B99] McKinnon P. J. (2013). Maintaining genome stability in the nervous system. *Nat. Neurosci.* 16 1523–1529. 10.1038/nn.3537 24165679 PMC4112580

[B100] McKinnon P. J. Caldecott K. W. (2007). DNA strand break repair and human genetic disease. *Annu. Rev. Genomics Hum. Genet.* 8 37–55. 10.1146/annurev.genom.7.080505.115648 17887919

[B101] Mejzini R. Flynn L. L. Pitout I. L. Fletcher S. Wilton S. D. Akkari P. A. (2019). ALS genetics, mechanisms, and therapeutics: where are we now? *Front. Neurosci.* 13:1310. 10.3389/fnins.2019.01310 31866818 PMC6909825

[B102] Mitchell J. Paul P. Chen H. J. Morris A. Payling M. Falchi M. (2010). Familial amyotrophic lateral sclerosis is associated with a mutation in D-amino acid oxidase. *Proc. Natl. Acad. Sci. U.S.A.* 107 7556–7561. 20368421 10.1073/pnas.0914128107 PMC2867752

[B103] Mitra J. Guerrero E. N. Hegde P. M. Liachko N. F. Wang H. Vasquez V. (2019). Motor neuron disease-associated loss of nuclear TDP-43 is linked to DNA double-strand break repair defects. *Proc. Natl. Acad. Sci. U.S.A.* 116 4696–4705. 10.1073/pnas.1818415116 30770445 PMC6410842

[B104] Naumann M. Pal A. Goswami A. Lojewski X. Japtok J. Vehlow A. (2018). Impaired DNA damage response signaling by FUS-NLS mutations leads to neurodegeneration and FUS aggregate formation. *Nat. Commun.* 9:335. 10.1038/s41467-017-02299-1 29362359 PMC5780468

[B105] Nguyen H. P. Van Mossevelde S. Dillen L. De Bleecker J. L. Moisse M. Van Damme P. (2018). NEK1 genetic variability in a Belgian cohort of ALS and ALS-FTD patients. *Neurobiol. Aging* 61 e1–e7. 10.1016/j.neurobiolaging.2017.08.021 28935222

[B106] Niblock M. Smith B. N. Lee Y. B. Sardone V. Topp S. Troakes C. (2016). Retention of hexanucleotide repeat-containing intron in C9orf72 mRNA: implications for the pathogenesis of ALS/FTD. *Acta Neuropathol. Commun.* 4:18. 10.1186/s40478-016-0289-4 26916632 PMC4766718

[B107] Nihei Y. Mori K. Werner G. Arzberger T. Zhou Q. Khosravi B. (2020). Poly-glycine-alanine exacerbates C9orf72 repeat expansion-mediated DNA damage via sequestration of phosphorylated ATM and loss of nuclear hnRNPA3. *Acta Neuropathol.* 139 99–118. 10.1007/s00401-019-02082-0 31642962 PMC6942035

[B108] Nissanka N. Moraes C. T. (2018). Mitochondrial DNA damage and reactive oxygen species in neurodegenerative disease. *FEBS Lett.* 592 728–742. 10.1002/1873-3468.12956 29281123 PMC6942696

[B109] Pardo B. Gomez-Gonzalez B. Aguilera A. (2009). DNA repair in mammalian cells: DNA double-strand break repair: how to fix a broken relationship. *Cell Mol. Life Sci.* 66 1039–1056. 10.1007/s00018-009-8740-3 19153654 PMC11131446

[B110] Pelegrini A. L. Moura D. J. Brenner B. L. Ledur P. F. Maques G. P. Henriques J. A. (2010). Nek1 silencing slows down DNA repair and blocks DNA damage-induced cell cycle arrest. *Mutagenesis* 25 447–454. 10.1093/mutage/geq026 20501547

[B111] Ravits J. Appel S. Baloh R. H. Barohn R. Rix Brooks B. Elman L. (2013). Deciphering amyotrophic lateral sclerosis: what phenotype, neuropathology and genetics are telling us about pathogenesis. *Amyotroph. Lateral Scler. Frontotemporal Degener.* 14(sup1) 5–18. 10.3109/21678421.2013.778548 23678876 PMC3779649

[B112] Rawal C. C. Zardoni L. Di Terlizzi M. Galati E. Brambati A. Lazzaro F. (2020). Senataxin ortholog sen1 limits DNA:RNA hybrid accumulation at DNA double-strand breaks to control end resection and repair fidelity. *Cell Rep.* 31:107603. 10.1016/j.celrep.2020.107603 32375052

[B113] Ray Chaudhuri A. Nussenzweig A. (2017). The multifaceted roles of PARP1 in DNA repair and chromatin remodelling. *Nat. Rev. Mol. Cell Biol.* 18 610–621. 10.1038/nrm.2017.53 28676700 PMC6591728

[B114] Riancho J. Castanedo-Vazquez D. Gil-Bea F. Tapia O. Arozamena J. Duran-Vian C. (2020). ALS-derived fibroblasts exhibit reduced proliferation rate, cytoplasmic TDP-43 aggregation and a higher susceptibility to DNA damage. *J. Neurol.* 267 1291–1299. 10.1007/s00415-020-09704-8 31938860

[B115] Robberecht W. Philips T. (2013). The changing scene of amyotrophic lateral sclerosis. *Nat. Rev. Neurosci.* 14 248–264. 10.1038/nrn3430 23463272

[B116] Robert C. Nagaria P. K. Pawar N. Adewuyi A. Gojo I. Meyers D. J. (2016). Histone deacetylase inhibitors decrease NHEJ both by acetylation of repair factors and trapping of PARP1 at DNA double-strand breaks in chromatin. *Leuk. Res.* 45 14–23. 10.1016/j.leukres.2016.03.007 27064363 PMC5007632

[B117] Rodriguez-Munoz M. Anglada T. Genesca A. (2022). A matter of wrapper: DEFECTS in the nuclear envelope of lagging and bridging chromatin threatens genome integrity. *Semin. Cell Dev. Biol.* 123 124–130. 10.1016/j.semcdb.2021.03.004 33757694

[B118] Rulten S. L. Rotheray A. Green R. L. Grundy G. J. Moore D. A. Gomez-Herreros F. (2014). PARP-1 dependent recruitment of the amyotrophic lateral sclerosis-associated protein FUS/TLS to sites of oxidative DNA damage. *Nucleic Acids Res.* 42 307–314. 10.1093/nar/gkt835 24049082 PMC3874156

[B119] Sartori A. A. Lukas C. Coates J. Mistrik M. Fu S. Bartek J. (2007). Human CtIP promotes DNA end resection. *Nature* 450 509–514. 17965729 10.1038/nature06337 PMC2409435

[B120] Sau D. De Biasi S. Vitellaro-Zuccarello L. Riso P. Guarnieri S. Porrini M. (2007). Mutation of SOD1 in ALS: a gain of a loss of function. *Hum. Mol. Genet.* 16 1604–1618. 10.1093/hmg/ddm110 17504823

[B121] Scully R. Panday A. Elango R. Willis N. A. (2019). DNA double-strand break repair-pathway choice in somatic mammalian cells. *Nat. Rev. Mol. Cell Biol.* 20 698–714. 10.1038/s41580-019-0152-0 31263220 PMC7315405

[B122] Sebastian R. Oberdoerffer P. (2017). Transcription-associated events affecting genomic integrity. *Philos. Trans. R. Soc. Lond. B Biol. Sci.* 372:20160288. 10.1098/rstb.2016.0288 28847825 PMC5577466

[B123] Shalom O. Shalva N. Altschuler Y. Motro B. (2008). The mammalian Nek1 kinase is involved in primary cilium formation. *FEBS Lett.* 582 1465–1470. 10.1016/j.febslet.2008.03.036 18387364

[B124] Stein D. Toiber D. (2017). DNA damage and neurodegeneration: the unusual suspect. *Neural Regen. Res.* 12 1441–1442. 10.4103/1673-5374.215254 29089988 PMC5649463

[B125] Stingele J. Bellelli R. Boulton S. J. (2017). Mechanisms of DNA-protein crosslink repair. *Nat. Rev. Mol. Cell Biol.* 18 563–573. 28655905 10.1038/nrm.2017.56

[B126] Sun Y. Curle A. J. Haider A. M. Balmus G. (2020). The role of DNA damage response in amyotrophic lateral sclerosis. *Essays Biochem.* 64 847–861. 10.1042/EBC20200002 33078197 PMC7588667

[B127] Thapa K. Khan H. Sharma U. Grewal A. K. Singh T. G. (2021). Poly (ADP-ribose) polymerase-1 as a promising drug target for neurodegenerative diseases. *Life Sci.* 267:118975. 10.1016/j.lfs.2020.118975 33387580

[B128] Thompson L. H. Schild D. (2001). Homologous recombinational repair of DNA ensures mammalian chromosome stability. *Mutat. Res.* 477 131–153. 10.1016/s0027-5107(01)00115-4 11376695

[B129] Torgovnick A. Schumacher B. (2015). DNA repair mechanisms in cancer development and therapy. *Front. Genet.* 6:157. 10.3389/fgene.2015.00157 25954303 PMC4407582

[B130] Tsang C. K. Liu Y. Thomas J. Zhang Y. Zheng X. F. (2014). Superoxide dismutase 1 acts as a nuclear transcription factor to regulate oxidative stress resistance. *Nat. Commun.* 5:3446. 10.1038/ncomms4446 24647101 PMC4678626

[B131] Tyzack G. E. Luisier R. Taha D. M. Neeves J. Modic M. Mitchell J. S. (2019). Widespread FUS mislocalization is a molecular hallmark of amyotrophic lateral sclerosis. *Brain* 142 2572–2580. 10.1093/brain/awz217 31368485 PMC6735815

[B132] van Blitterswijk M. DeJesus-Hernandez M. Niemantsverdriet E. Murray M. E. Heckman M. G. Diehl N. N. (2013). Association between repeat sizes and clinical and pathological characteristics in carriers of C9ORF72 repeat expansions (Xpansize-72): a cross-sectional cohort study. *Lancet Neurol.* 12 978–988. 10.1016/S1474-4422(13)70210-2 24011653 PMC3879782

[B133] van Rheenen W. Shatunov A. Dekker A. M. McLaughlin R. L. Diekstra F. P. Pulit S. L. (2016). Genome-wide association analyses identify new risk variants and the genetic architecture of amyotrophic lateral sclerosis. *Nat. Genet.* 48 1043–1048. 10.1038/ng.3622 27455348 PMC5556360

[B134] Vaz B. Halder S. Ramadan K. (2013). Role of p97/VCP (Cdc48) in genome stability. *Front. Genet.* 4:60. 10.3389/fgene.2013.00060 23641252 PMC3639377

[B135] Volkening K. Leystra-Lantz C. Yang W. Jaffee H. Strong M. J. (2009). Tar DNA binding protein of 43 kDa (TDP-43), 14-3-3 proteins and copper/zinc superoxide dismutase (SOD1) interact to modulate NFL mRNA stability. Implications for altered RNA processing in amyotrophic lateral sclerosis (ALS). *Brain Res.* 1305 168–182. 10.1016/j.brainres.2009.09.105 19815002

[B136] Walker C. Herranz-Martin S. Karyka E. Liao C. Lewis K. Elsayed W. (2017). C9orf72 expansion disrupts ATM-mediated chromosomal break repair. *Nat. Neurosci.* 20 1225–1235. 10.1038/nn.4604 28714954 PMC5578434

[B137] Wang H. Guo W. Mitra J. Hegde P. M. Vandoorne T. Eckelmann B. J. (2018). Mutant FUS causes DNA ligation defects to inhibit oxidative damage repair in Amyotrophic Lateral Sclerosis. *Nat. Commun.* 9:3683. 10.1038/s41467-018-06111-6 30206235 PMC6134028

[B138] Wang M. D. Gomes J. Cashman N. R. Little J. Krewski D. (2014). Intermediate CAG repeat expansion in the ATXN2 gene is a unique genetic risk factor for ALS–a systematic review and meta-analysis of observational studies. *PLoS One* 9:e105534. 10.1371/journal.pone.0105534 25148523 PMC4141758

[B139] Wang W. Y. Pan L. Su S. C. Quinn E. J. Sasaki M. Jimenez J. C. (2013). Interaction of FUS and HDAC1 regulates DNA damage response and repair in neurons. *Nat. Neurosci.* 16 1383–1391. 10.1038/nn.3514 24036913 PMC5564396

[B140] Wang X. D. Zhu M. W. Shan D. Wang S. Y. Yin X. Yang Y. Q. (2019). Spy1, a unique cell cycle regulator, alters viability in ALS motor neurons and cell lines in response to mutant SOD1-induced DNA damage. *DNA Repair. (Amst)* 74 51–62. 10.1016/j.dnarep.2018.12.005 30630676

[B141] Wang Y. Zhang N. Zhang L. Li R. Fu W. Ma K. (2016). Autophagy regulates chromatin ubiquitination in DNA damage response through elimination of SQSTM1/p62. *Mol. Cell* 63 34–48. 10.1016/j.molcel.2016.05.027 27345151

[B142] Williams K. L. Topp S. Yang S. Smith B. Fifita J. A. Warraich S. T. (2016). CCNF mutations in amyotrophic lateral sclerosis and frontotemporal dementia. *Nat. Commun.* 7:11253. 10.1038/ncomms11253 PMC4835537 27080313

[B143] Williams K. L. Warraich S. T. Yang S. Solski J. A. Fernando R. Rouleau G. A. (2012). UBQLN2/ubiquilin 2 mutation and pathology in familial amyotrophic lateral sclerosis. *Neurobiol. Aging* 33 2527 e3–e10. 10.1016/j.neurobiolaging.2012.05.008 22717235

[B144] Yeo A. J. Becherel O. J. Luff J. E. Graham M. E. Richard D. Lavin M. F. (2015). Senataxin controls meiotic silencing through ATR activation and chromatin remodeling. *Cell Discov.* 1:15025. 10.1038/celldisc.2015.25 27462424 PMC4860845

[B145] Yuan R. Liu Q. Segeren H. A. Yuniati L. Guardavaccaro D. Lebbink R. J. (2019). Cyclin F-dependent degradation of E2F7 is critical for DNA repair and G2-phase progression. *EMBO J.* 38:e101430. 10.15252/embj.2018101430 31475738 PMC6792010

[B146] Zhao B. Rothenberg E. Ramsden D. A. Lieber M. R. (2020). The molecular basis and disease relevance of non-homologous DNA end joining. *Nat. Rev. Mol. Cell Biol.* 21 765–781. 10.1038/s41580-020-00297-8 33077885 PMC8063501

[B147] Zhu C. Rogers A. Asleh K. Won J. Gao D. Leung S. (2020). Phospho-Ser(784)-VCP Is required for DNA damage response and is associated with poor prognosis of chemotherapy-treated breast cancer. *Cell Rep.* 31:107745. 10.1016/j.celrep.2020.107745 32521270 PMC7282751

[B148] Zou Z. Y. Zhou Z. R. Che C. H. Liu C. Y. He R. L. Huang H. P. (2017). Genetic epidemiology of amyotrophic lateral sclerosis: a systematic review and meta-analysis. *J. Neurol. Neurosurg. Psychiatry* 88 540–549. 10.1136/jnnp-2016-315018 28057713

